# Diagnosis and Management of Functional Pancreatic Neuroendocrine Tumors in Children—A Systematic Review

**DOI:** 10.3390/diagnostics15172176

**Published:** 2025-08-28

**Authors:** Dorotea Keretić, Marko Bašković

**Affiliations:** 1 Department of Pediatric Surgery, Children’s Hospital Zagreb, Ulica Vjekoslava Klaića 16, 10000 Zagreb, Croatia; dorotea.keretic@kdb.hr; 2 School of Medicine, University of Zagreb, Šalata 3, 10000 Zagreb, Croatia; 3 Scientific Centre of Excellence for Reproductive and Regenerative Medicine, School of Medicine, University of Zagreb, Šalata 3, 10000 Zagreb, Croatia; 4 School of Medicine, Catholic University of Croatia, Ilica 242, 10000 Zagreb, Croatia; 5 Croatian Academy of Medical Sciences, Kaptol 15, 10000 Zagreb, Croatia

**Keywords:** pancreatic neuroendocrine tumor, insulinoma, gastrinomas, glucagonoma, somatostatinoma, VIPoma, children, adolescents, pediatrics, pediatric surgery

## Abstract

**Background**: Functional pancreatic neuroendocrine tumors (FpNETs) are extremely rare in childhood and adolescence, with an incidence of less than 0.1 per million. Since there is currently no systematic review of the literature on FpNETs in children, this study aims to summarize findings from studies focusing on clinical characteristics, diagnostics, treatment modalities, and outcomes. **Methods**: A systematic review was conducted following the PRISMA guidelines. A literature search was performed using three electronic databases: PubMed, Scopus, and Web of Science. An age filter was used during the search to limit results to childhood and adolescence. There was no limit set in relation to the type and the language of the article. **Results**: Out of 80,742 records identified, 91 studies met the inclusion criteria and were included in the review. Two studies included patients with insulinoma and gastrinomas, that is, insulinomas and glucagonoma. Of the included studies, 71 were insulinomas, 10 were gastrinomas, 3 were glucagonomas, 6 were VIPomas, and 3 were mixed FpNETs. A total of 163 children with FpNETs were analyzed, with a median age of 12 years. A total of 48 cases were reported in childhood, while 115 cases were reported in adolescence. The results indicate that FpNETs were more prevalent in males. Almost all patients presented with symptoms appropriate to the type of tumor. A significant proportion of tumors were associated with MEN1. In almost all patients, the symptomatology was accompanied by elevated levels of specific hormones. US, CT, PET-CT, MRI, and EUS were the dominant imaging modalities. Surgical approaches and types of resections, depending on the type, association with the syndrome, location, and size of the tumor, were quite heterogeneous. Grade 1 and Grade 2 tumors were nearly equally represented. There was no recurrence in most patients. **Conclusions**: Early suspicion based on specific clinical symptomatology is essential for timely diagnosis. Accurate localization and size based on modern radiological diagnostics, accompanied by biochemical and genetic testing, are essential for optimal management. Adequate surgical resection offers the best chance of cure, with the lowest risk of recurrence. Additional multicenter registries and studies are needed in the future to better understand tumor behavior, optimal management, and outcomes of FpNETs.

## 1. Introduction

Although pancreatic neuroendocrine tumors (pNETs) are rare neoplasms in adults, they are extremely rare in childhood and adolescence, with an incidence of less than 0.1 per million [1,2]. They can be functional or nonfunctional, depending on whether they secrete hormones or not [3]. They can be sporadic or associated with specific hereditary syndromes such as multiple endocrine neoplasia type 1 (MEN1), von Hippel–Lindau (VHL) disease, neurofibromatosis type 1 (NF1), and tuberous sclerosis complex (TSC) [4]. As far as functional tumors are concerned in children, insulinomas predominate (50–60%), then gastrinomas (20–30%), followed by glucagonomas, VIPomas, and somatostatinomas (10%) [5].

Although most insulinomas are sporadic, some are associated with genetic syndromes. Most of them are solitary and localized in the pancreas. They are characterized by recurrent episodes of hypoglycemia with episodes of neuroglycopenic symptoms [6,7]. Most gastrinomas are also sporadic, and some may occur as part of a syndrome. Abdominal pain and diarrhea are the most common symptoms, and a large proportion of patients develop recurrent peptic ulcer disease [8]. The characteristic clinical features of glucagonoma, which is also usually solitary, result from excessive glucagon secretion and include hyperglycemia, progressive weight loss, normocytic anemia, and a characteristic skin condition known as necrolytic migratory erythema (NME) [9]. Pancreatic VIPomas are usually solitary, rarely as part of a syndrome, and occur in the tail of the pancreas in 75% of patients. They are characterized by profuse watery diarrhea with mild abdominal pain [10,11]. Most somatostatinomas are solitary within the head of the pancreas. The most common symptoms are abdominal pain and weight loss. Less commonly, patients present with somatostatinoma syndrome characterized by diabetes mellitus/glucose intolerance, cholelithiasis, and diarrhea/steatorrhea [12].

This systematic review aims to comprehensively analyze all cases of pediatric and adolescent patients with functional pNETs in order to systematically analyze clinical characteristics, diagnostic methods, treatment modalities, and outcomes, to optimize the management of functional pNETs in children.

## 2. Materials and Methods

### 2.1. Study Design and Search Strategy

A systematic review was performed according to Preferred Reporting Items for Systematic Reviews and Meta-Analysis (PRISMA) guidelines [13].

To identify the total number of articles of interest, we searched the electronic databases PubMed, Scopus, and Web of Science on 1 June 2025. Reading the articles and processing the data took one month. The search combinations used included the Boolean operators “AND” and “OR” in combination with the following MeSH and free text terms: [(pancrea*) OR (islet*) OR langerhans] AND [(endocrin*) OR (neuroendocrin*)] AND [(neoplasm*) OR (tumor*) OR (tumour*) OR (cancer*) OR (carcinom*) OR (adenom*)].

The Boolean logical operator expressions were used to search within databases, as follows:

PubMed: (“pancrea*”[All Fields] OR “islet*”[All Fields] OR (“langerhan”[All Fields] OR “langerhan’s”[All Fields] OR “langerhans”[All Fields] OR “langerhans’s”[All Fields])) AND (“endocrin*”[All Fields] OR “neuroendocrin*”[All Fields]) AND (“neoplasm*”[All Fields] OR “tumor*”[All Fields] OR “tumour*”[All Fields] OR “cancer*”[All Fields] OR “carcinom*”[All Fields] OR “adenom*”[All Fields]).

Scopus: TITLE-ABS-KEY (((pancrea*) OR (islet*) OR langerhans) AND ((endocrin*) OR (neuroendocrin*)) AND ((neoplasm*) OR (tumor*) OR (tumour*) OR (cancer*) OR (carcinom*) OR (adenom*))).

Web of Science (editions: A&HCI, BKCI-SSH, BKCI-S, CCR-EXPANDED, ESCI, IC, CPCI-SSH, CPCI-S, SCI-EXPANDED, SSCI): ((pancrea*) OR (islet*) OR langerhans) AND ((endocrin*) OR (neuroendocrin*)) AND ((neoplasm*) OR (tumor*) OR (tumour*)OR (cancer*) OR (carcinom*) OR (adenom*)) (All Fields).

**Figure 1:**
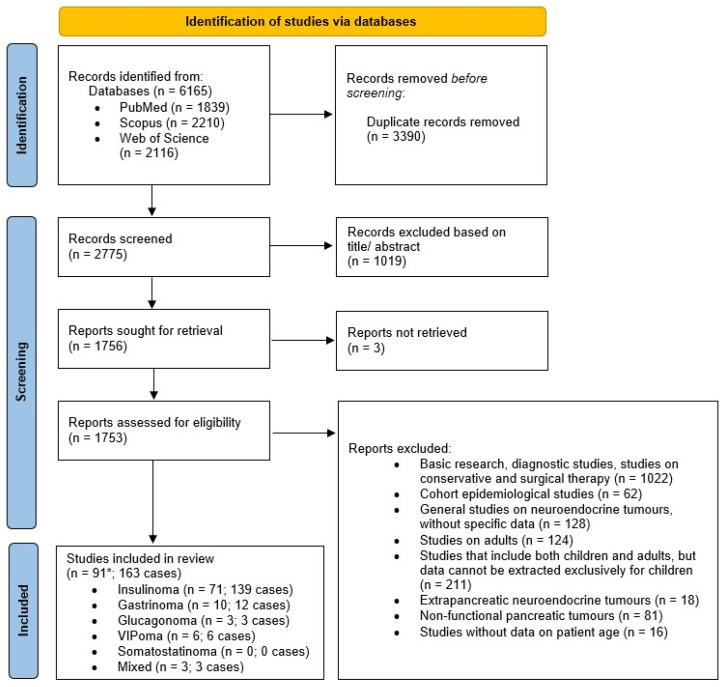
PRISMA flow diagram. * Two studies included patients with insulinoma and gastrinomas (Goudet et al., 2015) [39], and insulinomas and glucagonoma (van den Akker et al., 2012) [49], respectively.

An age filter was used during the search to limit results to children and adolescents. There was no limit set in relation to the type or the language of the article. No text analysis tools were used. The study selection process is described in Figure 1.

### 2.2. Inclusion and Exclusion Criteria

To be included, the report had to contain data on a patient aged between 0 and 18 years who was treated for a functional pancreatic neuroendocrine tumor. Articles that reported on cases older than 18 years were excluded, while for articles that simultaneously included patients younger than and older than 18 years of age, only patients younger than 18 years were considered. The following information of interest was sought within the report: type of tumor, patient’s age, patient’s gender, reason for hospital visit (clinical presentation), laboratory findings, genetic analysis (possible pNET within the syndrome), radiological findings, tumor location, tumor size, type of treatment, histopathological findings, and outcome. If a study contained less than one-third of the requested information, it was not considered.

### 2.3. Screening Process, Critical Appraisal, and Data Extraction

After removing duplicate records, studies were selected in a four-step process. The first step was to assess their eligibility based on the title and abstract, which were independently reviewed by two investigators (D.K. and M.B.). In the second step, the investigators reviewed the articles that met the predefined inclusion and exclusion criteria in the full text. In case of disagreement, a consensus was reached by discussion. Subsequently, the aforementioned data were extracted by the investigators (see the flow diagram summarizing the selection of studies for inclusion in the systematic review). The review protocol was registered with the International Prospective Register of Systematic Reviews (PROSPERO, CRD420251104371).

### 2.4. Assessment of the Methodological Quality and the Risk of Bias of Studies

Depending on the type of study, methodological quality and potential sources of bias in the included studies were independently assessed by D.K. and M.B. using the Joanna Briggs Institute (JBI) Critical Appraisal Checklist [14] (Table S1). Disagreements between the investigators at various stages of the review were resolved through discussion. For scoring, each “Yes” response was awarded one point, while “No,” “Unclear,” and “Not applicable” responses received zero points. The total score was determined by summing the points from all “Yes” responses and was then converted into a percentage by dividing by the maximum possible score. Based on this percentage, the methodological quality of each study was classified as low (<50%), moderate (50–74%), or high (>75%).

### 2.5. Statistical Analysis

The obtained data were analyzed using the Microsoft Excel^®^ software program (XLSTAT^®^) for Windows, version 2020.5.1 (Microsoft Corporation, Redmond, WA, USA). Categorical variables were expressed in absolute numbers and percentages. Continuous variables were expressed as mean with standard deviation (SD) and median (Mdn) with interquartile range (IQR) as appropriate. The proportions are shown in percentages, while the existence of a difference between groups of interest was tested with the chi-square test. A significance level of 0.05 was used.

## 3. Results

### 3.1. Study Selection

Based on the aforementioned search strategy, a total of 80,742 records were identified by searching the PubMed (24,619), Scopus (28,758), and Web of Science (27,365) databases. Using filters for childhood and adolescence, a total of 6165 records remained, of which 3390 were duplicates that were removed before the screening phase. Based on titles and abstracts, 1019 records were excluded during the screening phase. Of the remaining 1756 articles, 3 were not retrieved, and 1662 were excluded. Based on the inclusion and exclusion criteria, finally, 91 studies, with a total of 163 cases, were included in the systematic review. Two studies included patients with insulinoma and gastrinomas, that is, insulinomas and glucagonoma. Of the 91 studies, 71 were related to insulinomas, 10 to gastrinomas, 3 to glucagonomas, 6 to VIPomas, and 3 to mixed functional pNETs. All included studies were case reports or original studies of retrospective or prospective design. No cases of somatostatinomas were found in childhood or adolescence. The PRISMA flow diagram of the literature search is presented in Figure 1.

### 3.2. Study Characteristics, Risk of Bias, and Summary of Included Studies

Upon assessing the methodological quality and risk of bias using the JBI Critical Appraisal Checklist for Case Reports and Case Series, 86 studies were classified as high quality and 5 as medium quality, based on the overall quality assessment score (Table S1).

Gender data were available for 158 participants, of whom 90 (57%) were male and 68 (43%) were female. The median age was 12 years (range 30 h–17 years). There were 48 cases in childhood (<10 years), while 115 cases were reported in adolescence (≥10 years). The main characteristics of the studies included in this systematic review are shown in Table 1, Table 2, Table 3, Table 4 and Table 5.

**Table 1:** Articles included in the systematic review—insulinomas.

Study	Country	Type of Study	Type of Tumor	Patient’s Gender	Patient’s Age (Years)	Clinical Presentation	Laboratory Findings	Genetic Analysis	Radiological Modality	Tumor Location	Tumor Size (cm)	Type of Treatment	Histopathology	Outcome
Sales et al., 2025 [15]	Brazil	Case report	I	M	11	Generalized tonic–clonic seizures, hypoglycemia	Fasting glucose—33 mg/dL, insulin—12.1 mU/L	MEN1	MR	-	1 × 0.8	Surgical resection	-	Glycemic normalization
Murray et al. 2025 [16]	USA	Case report	I	M	13	Severe weakness, altered mental status, syncopal episodes	Glucose—98 mg/dL, insulin—44 μIU/mL	MEN1	CT + MR	Head + body + tail	0.3, 0,4, 0.7, 2.1	Surgical enucleation	NET G1/G2	Glycemic normalization
Lemus-Zepeda et al. 2025 [17]	Colombia	Case report	I	M	8	Hypoglycemia during an episode of abnormal movements	Glucose—33.5 mg/dL, insulin—13.3 µUI/mL	MEN1	MR	Uncinate process	2.7 × 2.2 × 1.6	Surgical enucleation	-	Glycemic normalization
Huang et al., 2024 [18]	China	Case report	I	M	14	Recurrent seizure-like episodes	Glucose—2.09 mmol/L, insulin—23.96 μU/mL	MEN1	MR	Body	1.7 × 1.2	Distal partial pancreatectomy	NET G2	Glycemic normalization
Kamińska-Jackowiak et al., 2024 [19]	Poland	Case report	I	F	15	Neuroglycopenia	Glucose—47 mg/dL, insulin—43 μUI/mL	MEN1	US + CT + MR	Head, tail	-	Enucleation	-	Reoperation
Moszczyńska et al., 2024 [20]	Poland	Case report	I	F	13	Weakness, paleness, profuse sweating, balance impairment, hypoglycemia	Glucose—27 mg/dL, insulin—90.9 μIU/mL	-	CT	Head	+liver mts	Modified Whipple’s operation	NET G2	Somatostatin analogs + allogeneic liver transplantation + relaparotomy
Tian et al., 2024 [21]	China	Original research	I	F	9	Dizziness	Glucose—1.8 mmol/L, insulin—129 pmol/L	-	US + MR	Head	3	Tumor enucleation	NET G1	glycemic normalization
				M	7	Dizziness	Glucose—3.9 mmol/L, insulin—1736 pmol/L		US + CT	Tail	1.5	Partial resection	NET G2	Glycemic normalization
				M	9	Dizziness	Glucose—0.5 mmol/L, insulin—69.3 pmol/L		US + MR	Neck	2	Tumor enucleation	NET G2	Glycemic normalization
				M	4	Seizure	Glucose—1.5 mmol/L, insulin—94.2 pmol/L		US + MR	Body	1.5	Partial resection	NET G1	Glycemic normalization
				M	4	Dizziness	Glucose—2.2 mmol/L, insulin—219.2 pmol/L		US + MR	Head	0.8	Subtotal resection	NET G1	Glycemic normalization, postoperative Creon
Vinhosa Bastos et al., 2023 [22]	Brazil	Case report	I	M	16	Episodes of muscle spasms in thighs and legs	Glucose—71 mg/dL	-	US + MR	Body–tail transition	2.2 × 1.3	Pancreatic nodulectomy	NET G2	Glycemic normalization, mild chronic neurogenic changes
Melikyan et al., 2023 [6]	Russia	Original research	I	M:F/9:13	11	-	Glucose—1.9 (0.5–2.2) mmol/L, insulin—20.9 (8.13–149) U/L	MEN1	US (7), CT and/or MR (18)	Head + tail	1.2, 0.3	Partial resection	NET G2	Pituitary adenoma at 13 years, hPTH at 15 years
					8			MEN1		Head	2.5	Subtotal pancreatectomy	-	hPRL at 21 years (on cabergoline), gastrinoma at 25 years
					13			MEN1		Tail	3	Enucleation	-	hPTH, hPRL, adrenal nodular hyperplasia at 19 years
					11			MEN1		Body + tail	1.5, 1.4	Enucleation	NET G2	hPTH at 13 years, hPRL at 16 tears (on cabergoline)
					9			MEN1		Head + body + tail	2, 0.5, 0.6	Partial resection	NET G1	Somatotropinoma at 12 years, pNET at 13 years, hPTH at 14 years
					14			MEN1		Head + tail	3.7, 0.6	Enucleation	NET G2	hPTH at 18 years
					8			MEN1		Tail	1.1	Partial resection	NET G2	Glycemic normalization
					12			MEN1		Tail	2.3	Enucleation	NET G2	Glycemic normalization
					17			-		Tail	3.5	Enucleation	NET G2	Glycemic normalization
					13			-		Tail	3	Enucleation	NET G1	Glycemic normalization
					12			-		Body	1.5	Enucleation	NET G2	Glycemic normalization
					11			-		Body	6 + liver mts	Subtotal pancreatectomy + splenectomy	NET G2 in tumor, G3 in mts	Deceased at 11 years 8 months
					8			-		Tail	0.95	Enucleation	NET G1	-
					15			-		Head	1.56	Pancreatic resection	NET G1	-
					14			-		Head	2	Partial resection	NET G1	Creon
					13			-		Head	1.5	Partial resection	NET G2	Glycemic normalization
					16			-		Tail	1.1	Partial resection	NET G1	-
					14			-		Head	1.9	Enucleation	-	Epilepsy at 18 years
					11			-		Body	1	Enucleation	-	Glycemic normalization
					11			-		Head + body	1.5, 0.5, 1, 3	Enucleation at 11 years, partial resection at 12 years, pancreato-gastro-duodenal resection at 13 years	NET G1/G2	Postoperative DM, liver mts at 21 years, nephropathy at 32 years
					9			-		Body	1.9	Enucleation	NET G1	-
					17			-		Body	2.5	Partial resection	-	-
Shariq et al., 2022 [23]	United Kingdom	Original research	I	F	15	-	-	MEN1	EUS + CT + MR	Tail	6	Distal pancreatectomy	-	No recurrence of hypoglycemia
				F	10			MEN1	EUS + CT + MR	Tail	1.7	Distal pancreatectomy	NET G2	No recurrence of hypoglycemia
				M	10			MEN1	EUS + CT + MR	Tail	1	Distal pancreatectomy	-	No recurrence of hypoglycemia
				M	6			MEN1	EUS + CT + MR	Body	0.4	Distal pancreatectomy	NET G1	No recurrence of hypoglycemia
				F	16			MEN1	EUS + CT + MR	Tail	2.1	Distal pancreatectomy	NET G2	No recurrence of hypoglycemia
Sherafati et al., 2022 [24]	Iran	Case report	I	M	16	Generalized tonic–clonic seizures	Glucose—60 mg/dL, insulin—30.1 mIU/L	-	PET CT	Tail	1	Subtotal pancreatectomy	-	No recurrence
Yu et al., 2021 [25]	China	Case report	I	F	14	Increased daytime, walked unsteadily, twitching of the limbs	Glucose—1.9 mmol/L, insulin—23.2 μIU/mL	-	EUS + CT + MR	Head	1	Surgical enucleation	-	Glycemic normalization
Schulte Am Esch et al., 2021 [26]	Germany	Case report	I	M	10	Absence-like condition	Glucose—46 mg/dL, insulin—7.2 µU/L	MEN1	EUS + PET MR	Body	1.5	Robotic enucleation	NET G2	Glycemic normalization
Mahdi et al., 2020 [27]	Saudi Arabia	Case report	I	M	8	Multiple hypoglycemic attacks	Glucose—1.9 mmol/L, insulin—20 μU/mL	-	CT	Tail	1.2	Laparoscopic enucleation	NET G1	No recurrence
Al Azmi et al., 2020 [28]	Saudi Arabia	Case report	I	M	7	Hypoglycemia episodes	Glucose—1.9 mmol/L, insulin—20 µU/mL	-	CT + MR	Tail	2.5 × 1	Laparoscopic enucleation	-	No recurrence
Selberherr et al., 2019 [29]	Austria	Original research	I	M	15	Hypoglycemia	-	MEN1	EUS + CT + MR	Body + tail	2, 1.5, 0.6, 0.5	Left pancreatic resection	NET G2	Glycemic normalization
Escartín et al., 2018 [30]	Spain	Case report	I	M	11	Episode of fainting	Glucose—42 mg/dL, insulin—10.6 µU/mL	-	EUS + CT	Body–tail transition	1.1	Laparoscopic partial pancreatectomy	-	Glycemic normalization
Liang et al., 2018 [31]	China	Case report	I	F	9	Loss of consciousness, palpitations, convulsions	Glucose—2.2 mmol/L, insulin—15.35 μIU/mL	MEN1	PET CT + MR	Tail	1.1 × 1.3	Robotic enucleation	NET G2	No recurrence
Nakano et al., 2018 [32]	Japan	Case report	I	F	14	Convulsions	Glucose—33 mg/dL, insulin—11.2 µIU/mL	MEN1	MR	-	1	Surgical resection	-	-
Hu et al., 2017 [33]	China	Case report	I	F	9	Episodic fainting attacks with sweatiness	Glucose—1.84 mmol/L, insulin—8.2 mU/L	-	MR	Tail	2 × 2 × 1.2	Robot-assisted, distal pancreatectomy	NET G2	Glycemic normalization
Beisang et al., 2017 [34]	USA	Case report	I	M	16	Limb shaking	Glucose—41 mg/dL, insulin—21 uU/mL	-	EUS + MR	Uncinate process	1.6 × 1	Pancreaticoduodenectomy	-	Glycemic normalization
Yao et al., 2017 [35]	China	Original research	I	F	17	Hypoglycemia, somnolence	Insulin—25.7 mU/L	-	-	Tail	0.5	Enucleation	-	PNET recurrence—reenucleation
				M	14	Hypoglycemia	Insulin—19.7 mU/L		CT + MR	Head	7 + liver mts	Enucleation		PNET recurrence—pancreaticoduodenectomy
				F	15	Confusion	Insulin—7.9 mU/L		-	Uncinate process	2	Radical surgery		No recurrence
Kwon et al., 2016 [36]	Korea	Case report	I	F	9	Sudden loss of balance, tremor, generalized tonic–clonic seizure	Glucose—34 mg/dL, insulin—142.7 uIU/mL	MEN1	MR	Head	1.3 × 1.5	Enucleation	-	Glycemic normalization
Miron et al., 2016 [37]	Romania	Case report	I	M	11	Diffuse abdominal pain, cold sweats, confusion, tremor, paresthesias	Glucose 14—38 mg/dL, insulin—12.6 μU/mL	-	US + MR	Tail	1	Enucleation	-	Glycemic normalization
Vasikasin et al., 2016 [38]	Thailand	Case report	I	M	15	Lightheadedness, diaphoresis, palpitations	Glucose—1.5 mmol/L, insulin—13.34 μU/mL	-	CT	Tail	12.5 × 10 × 8.3	Distal pancreatectomy	.	Glycemic normalization
Goudet et al., 2015 [39]	France	Original research	I	F	5	Confusion	-	MEN1	-	-	1.3	Left pancreatectomy	-	No recurrence
Smith et al., 2015 [40]	USA	Case report	I	F	14	Unusual behaviors	Glucose—44 mg/dL, insulin—104 pmol/L	-	US + CT + MR + ASVS	Head/neck	2.1 × 1.3 × 0.8	Enucleation	NET G2	Glycemic normalization
Abu-Zaid et al., 2014 [41]	Saudi Arabia	Case report	I	M	10	Tremulousness, diaphoresis, increased hunger, confusion, fainting	Glucose—64 mg/dL, insulin—6 μU/mL	-	CT	Body	2.3 × 1.6 × 1.1	Enucleation	NET G2	Glycemic normalization
Padidela et al., 2014 [42]	United Kingdom	Original research	I	F	3	Neurological symptoms of hypoglycemia	Glucose—1.9 mmol/L, insulin—5.7 mIU/L	-	PET CT	Uncinate process	1	Enucleation	-	Glycemic normalization
				M	5		glucose—2.2 mmol/L, insulin—4.5 mIU/L	MEN1	MR	Head	1	Subtotal pancreatectomy		
				M	8		Glucose—1.5 mmol/L, insulin—5.8 mIU/L	MEN1	PET CT + MR	Uncinate process + tail	1.5	Partial pancreatectomy		
				F	8		Glucose—1.1 mmol/L, insulin—66.3 mIU/L	-	MR	Head	1.5	Subtotal pancreatectomy		
				M	8		Glucose—0.8 mmol/L, insulin—85 mIU/L	-	PET CT + MR	Tail	1.2	Partial pancreatectomy		
				F	11		Glucose—1.6 mmol/L, insulin—9 mIU/L	-	MR	Head	1.2	Enucleation		
				F	13		Glucose—2 mmol/L, insulin—7 mIU/L	-	MR	Uncinate process	0.8	Enucleation + pancreaticojejunostomy		
				F	13		Glucose—2.1 mmol/L, insulin—44.6 mIU/L	-	PET CT + MR	Uncinate process	2	Enucleation + pancreaticojejunostomy		
				M	15		Glucose—1.9 mmol/L, insulin—14.4 mIU/L	-	MR	Head	1.2	Subtotal pancreatectomy		
Gozzi Graf et al., 2014 [43]	Switzerland	Case report	I	M	14	Seizures	Glucose—2.9 mmol/L, insulin—47.9 pmol/L	MEN1	MR	Tail	2.5	Laparoscopic distal pancreatectomy	-	No recurrence
				M	11	Dizziness, disorientation, vomiting	Glucose—2.6 mmol/L, insulin—93.3 pmol/L	-	PET CT + MR	Head	1.2	Enucleation	-	No recurrence
Peranteau et al., 2013 [44]	USA	Original research	I	F	4	-	-	-	CT + ASVS	Head	0.7	Enucleation	-	Glycemic normalization
				M	8			-	EUS + PET CT + MR	Tail	1.5	Enucleation		
				M	9			-	EUS + CT + MR	Head	1.5	Enucleation		
				M	11			-	US + CT	Tail	1.5	Distal pancreatectomy		
				F	5			-	PET CT	Tail	1.2	Distal pancreatectomy		
				M	14			-	EUS + CT	Neck	0.7	Enucleation		
				M	11			MEN1	MR	Head, body, tail	0.3	Distal pancreatectomy + enucleation		
Horváth et al., 2013 [45]	Romania	Case report	I	M	16	Confusion, generalized tonic–clonic seizures	Glucose—25 mg/dL, insulin—23.8 μU/mL	-	CT	Tail	1.6	Enucleation	-	No recurrence
Bartsch et al., 2013 [46]	Germany	Original research	I	M	12	Hypoglycemia-related symptoms	I/G ratio > 0.3	MEN1	EUS or CT or MR	-	1.7	Enucleation	NET G1	No recurrence, dead of unrelated cause
				M	9			MEN1		-	1.3	Subtotal distal pancreatectomy	NET G1	No recurrence
				F	15			MEN1		-	4	Enucleation	NET G1	No recurrence
				F	11			MEN1		-	0.8	Enucleation	-	No recurrence
Ahmed et al., 2013 [47]	Canada	Original research	I	F	-	Shakiness, seizures, hypoglycemia	-	-	US + MR	Tail	2.2	Resection	-	-
Toaiari et al., 2013 [48]	Italy	Original research	I	M	17	-	-	-	-	-	1.3	-	NET G1	-
				F	15			MEN1			2.5		NET G2	
				M	17			-			3.3		NET G1	
van den Akker et al., 2012 [49]	Belgium	Original research	I	F	15	-	-	-	-	-	4	Distal pancreatectomy	-	No recurrence
				F	13						2	Distal pancreatectomy		No recurrence
				M	8						0.9	Distal pancreatectomy		No recurrence
de Paiva et al., 2012 [50]	Brazil	Case report	I	M	17	Tonic–clonic generalized seizure	Glucose—17 mg/dL	MEN1	MR	-	-	Partial pancreatectomy	-	No recurrence
Marchegiani et al., 2011 [51]	Italy	Original research	I	F	15	Asymptomatic (incidental diagnosis)	-	MEN1	-	Head, body	2.5	Enucleation	NET G1	No recurrence
Fabbri et al., 2010 [52]	Brazil	Case report	I	M	8	Sweating, palpitation, tremulousness, hunger, anxiety	Glucose—19 mg/dL, insulin—26.2 IU/mL	MEN1	US + CT	Body	0.6, 0.3	Partial pancreatectomy	-	No recurrence
Janem et al., 2010 [53]	Jordan	Case report	I	M	12	Abdominal pain, weight loss, generalized weakness	Glucose—25 mg/dL, insulin—55.7 mIU/mL	-	CT	-	Liver, bone, bone marrow mts	Chemotherapy	-	Died
Ozen et al., 2009 [54]	Turkey	Case report	I	M	16	Generalized tonic–clonic seizure	Glucose—20 mg/dL, insulin—8.8 IU/L	-	EUS + CT	Tail	0.8	Resection	-	No recurrence
Concolino et al., 2008 [55]	Italy	Case report	I	F	15	Episodes of absences, behavioral disturbances, seizures	Glucose—576.6 mmol/L, insulin—4.5 pmol/L	MEN1	MR	Head, body, tail	2 × 1.8, 0.8, 0.85	Enucleation	-	-
Bonfig et al., 2007 [56]	Germany	Case report	I	M	15	Hypoglycemic seizures	Glucose—41 mg/dL, insulin—15.6 μU/mL	-	EUS + PET CT + MR	Tail	1 × 1.5	Laparoscopic enucleation	-	Glycemic normalization
Blasetti et al., 2007 [57]	Italy	Case report	I	F	17	Refractory seizures	Glucose—1.9 mM/L, insulin—22.1 μΙΙ/mL	-	CT	Tail	1.5	Resection	-	No recurrence
Nakagawa et al., 2007 [58]	Japan	Case report	I	F	9	Generalized convulsion	Glucose—32 mg/dL, insulin—13 μU/mL	-	CT + MR + ASVS	Head	4 × 3.5 × 3.3	Enucleation	-	No recurrence
de Vogelaere et al., 2006 [59]	Belgium	Case report	I	M	8	Episodes of absences, headache, visual and auditive disturbances	Insulin > 10 μU/mL	MEN1	US + CT + MR	Body	1 × 1.5	Laparoscopic enucleation	-	No recurrence
Karachaliou et al., 2006 [60]	Greece	Case report	I	F	10	Pallor, sweating, weakness, loss of consciousness, convulsions	Glucose—2.05 mmol/L, insulin—12.67 μU/mL	-	CT + MR	Tail	2	Distal pancreatectomy	-	Glycemic normalization
Zografos et al., 2005 [61]	Greece	Case report	I	F	17	Loss of consciousness	Glucose—1.6 to 3 mmol/L, insulin > 6 iu/L	-	EUS + CT + MR	Tail	0.98 × 0.82	enucleation	-	no recurrence
Kann et al., 2005 [62]	Germany	Case report	I	M	15	Severe hypoglycemia, multiple convulsions	-	-	EUS	Tail	1.5	Laparoscopic enucleation	-	No recurrence
Langer et al., 2005 [63]	Germany	Case report	I	M	15	Tonic–clonic convulsions, tremor, hunger	Glucose—28 mg/dL, insulin—15.9 µU/L	-	EUS + CT + MR	Tail	0.8 × 1.3	Laparoscopic enucleation	-	No recurrence
Ardengh et al., 2004 [64]	Brazil	Original research	I	M	14	-	-	-	EUS + CT	Tail	2.2	Distal pancreatectomy	-	-
Van Nieuwenhove et al., 2003 [65]	Belgium	Original research	I	M	8	Hypoglycemia	-	MEN1	MR	Neck	>1	Enucleation	-	No recurrence
Lo et al., 2003 [66]	Hong Kong	Case report	I	M	13	Fainting attacks, dizziness, sweatiness, decreased consciousness	Glucose—2 mmol/L, insulin—20 mIU/L	-	EUS + CT + MR	Tail	2.3	Laparoscopic distal pancreatectomy	-	No recurrence
Hussain et al., 2002 [67]	United Kingdom	Case report	I	F	8	Tonic–clonic seizures	Glucose—1.9 mmol/L, insulin—37.2 pmol/L	-	MR (-)	Head body transition	1.5	Subtotal pancreatectomy	-	No recurrence
Nollet et al., 2001 [68]	Belgium	Case report	I	M	16	Muscle fatigue and moderate paresthesia	Glucose—2.4 mmol/L, insulin—48 mU/L	-	US + CT + MR	Tail	2.5 × 3	Distal pancreatectomy	-	No recurrence
Chavan et al., 2000 [69]	Germany	Original research	I	M	11	-	-	MEN1	US + CT + MR + ASVS	Tail	1, 0.8	-	-	No recurrence
Proye et al., 1998 [70]	France	Original research	I	F	16	-	-	-	EUS	Head	1.4	Enucleation	-	No recurrence
				M	12				EUS	Distal pancreas	1	Enucleation		No recurrence
Beccaria et al., 1997 [71]	Italy	Case report	I	M	11	Loss of consciousness, vertigo, amaurosis, ataxic gait	Glucose—1.7 mmol/L, insulin—24 mU/L;	-	US + MR	Body, tail	2 × 1.4, 0.6, 2 × 1, 0.2	Enucleation	-	Glycemic normalization
Waeber et al., 1997 [72]	Switzerland	Case report	I	M	17	Episodes of sudden absence, inappropriate response to verbal stimuli, somnolence	Glucose—1.5 mmol/L, insulin 51 mU/L	MEN1	EUS + CT	Head	1.5	Enucleation	-	No recurrence
Maioli et al., 1992 [73]	Italy	Case report	I	M	14	Loss of consciousness, sweating	Glucose/insulin ratio < 3	-	US + CT	Tail	1.5	Partial pancreatectomy—reoperation	-	No recurrence
				F	6	Vertigo	-	-	-	Tail	1	Enucleation	-	No recurrence
Winocour et al., 1992 [74]	United Kingdom	Case report	I	F	15	Reversible neurological disturbances	Glucose—1.5 mmol/L, insulin—245 pmol/L	MEN1	CT	Head, body, tail	1.5 × 1 × 0.5	Sub-total pancreatectomy, pancreaticoduodenectomy (reoperation)	-	Residual insulinoma, death
Telander et al., 1986 [75]	USA	Original research	I	M	15	-	-	MEN1	US	Head, body	1.5, 0.5, 0.5, 0.2	85% pancreatectomy + enucleation	-	-
				F	13			MEN1	US	Tail	0.2–0.9	85% pancreatectomy		
				M	14			-	US + CT	Tail	2.5	Distal pancreatectomy		
				M	16			-	US + CT	Head	1.6	85% pancreatectomy + enucleation		
				M	12			-	US	Head	1.5	85% pancreatectomy + enucleation		
Rasbach et al., 1985 [76]	USA	Original research	I	-	10	Dizziness, weakness, blurred vision, confusion, headaches, seizures	-	MEN1	Angiography	-	0.4–3	Enucleation	-	Recurrent hyperinsulinism—reoperation
					10			MEN1	Angiography		2.7	Resection (80%)		Persistent hyperinsulinism—reoperation
					17			MEN1	Angiography		0.3–2.3	Resection (85%)		No recurrence
					14			MEN1	US		0.2–2.4	Resection (85%)		No recurrence
					12			MEN1	US		0.2–0.9	Resection (85%)		Persistent hyperinsulinism
Stringel et al., 1985 [77]	USA	Case report	I	M	12	Seizures, pallor, clammy skin, confusion, tachycardia	Glucose—2 mmol/L	-	US + CT + angiography	Body	-	Distal pancreatectomy	-	No recurrence
Gough, 1984 [78]	United Kingdom	Case report	I	M	30 h	Jittery, convulsion	-	-	-	Neck	0.5	Enucleation	-	No recurrence
				F	9	Fainting attacks with convulsions			Angiography	Tail	1	Distal pancreatectomy		No recurrence
MacDonald et al., 1983 [79]	USA	Case report	I	F	2 months	Listlessness, tremulousness, eye rolling, weak suck	Glucose < 30 mg/dL, insulin—29 µU/mL	-	-	-	-	Pancreatectomy (50%)	-	No recurrence
				F	2 months	Generalized seizures	Glucose—30 mg/dL, insulin—49 µU/mL			Head	1	Pancreatectomy (65%)		No recurrence
Bordi et al., 1982 [80]	Italy	Case report	I	M	3 days	Convulsions	Glucose—0.3 mmol/L, insulin >12 µU/mL	-	-	Body	0.4	Pancreatectomy (80%)	-	No recurrence
Glickman et al., 1980 [81]	USA	Original research	I	M	9	Convulsions	Glucose—5 mg/100 mL	-	-	-	1	Enucleation	-	Recurrent 10 cm adenoma. died after resection
				M	16	Seizure	Glucose—40 mg/100 mL		Angiography	Head, neck	2	Enucleation		No recurrence
				M	7	Fainting, seizure	Glucose—35 mg/100 mL		-	Body	1.5	Enucleation		No recurrence
				F	5 days	Seizure	Glucose—4 mg/100 mL		-	Head	0.3	Pancreatectomy (85%)		No recurrence
Ginsberg-Fellner et al., 1980 [82]	USA	Case report	I	M	8	Recurring seizures	Glucose—25 mg/dL, insulin—96 µU/mL	-	Angiography	Body	1.5	Enucleation	-	No recurrence
Cameron et al., 1972 [83]	Canada	Case report	I	M	6	Unconsciousness, irritable	Glucose—56 mg/100 mL	-	Angiography	Body tail transition	2 × 1.5	Enucleation	-	No recurrence
Heitz et al., 1971 [84]	Switzerland	Case report	I	M	12	Hypoglycemic attacks	Glucose—27 mg/100 mL, insulin—150 μU/mL	-	Angiography + US	Head	2 × 1.5 × 1	Enucleation	-	No recurrence

I—insulinoma, M—male, F—female, US—ultrasound, EUS—endoscopic ultrasound, MR—magnetic resonance, CT—computed tomography, ASVS—arterial stimulation with venous sampling, PET—positron emission tomography, NET—neuroendocrine tumor, PNET—pancreatic neuroendocrine tumor, G—gradus, hPTH—hyperparathyroidism, hPRL—hyperprolactinemia, DM—diabetes mellitus, MEN1—multiple endocrine neoplasia type 1, mts—metastases.

**Table 2:** Articles included in the systematic review—gastrinomas.

Study	Country	Type of Study	Type of Tumor	Patient’s Gender	Patient’s Age (Years)	Clinical Presentation	Laboratory Findings	Genetic Analysis	Radiological Modality	Tumor Location	Tumor Size (cm)	Type of Treatment	Histopathology	Outcome
Nath et al., 2017 [8]	India	Case report	G	M	12	Epigastric pain, vomiting, occasional loose stools, peptic ulcer	Gastrin—940 pg/mL	-	US + CT	Head	3.8 × 2.8	Excised in toto	-	No recurrence
Goyal et al., 2016 [85]	India	Case report	G	M	12	Abdominal pain, diarrhea, vomiting, weight loss, peptic ulcer	Gastrin > 8000 pg/mL	-	US + CT	Head	3.3 × 2.3 + liver mts	-	-	Oncology follow-up
Murase et al., 2015 [86]	Japan	Case report	G	M	9	Vomiting, peptic ulcer	Gastrin—834 pg/mL	-	US + CT + MR	Head	-	Laparoscopic-assisted Pancreaticoduodenectomy	-	No recurrence
Goudet et al., 2015 [39]	France	Original research	G	F	6	Diarrhea, esophagitis, multiple ulcerations of duodenum and antral stomach	Gastrin—3000 pg/mL	MEN1	US	-	0.2	-	-	Reoperated on at 14, 18, and 19 yr old for local recurrence
				M	16	Abdominal pain, intermittent vomiting episodes, esophagitis, multiple duodenal ulcers	Gastrin—870 pg/mL	MEN1	-	-	-	-		-
				F	17	Abdominal pain, heart burn, diarrhea, esophagitis, multiple gastrointestinal ulcers	Gastrin—610 pg/mL	MEN1	-	Head + tail	-	Enucleation + left pancreatectomy-		-
Massaro et al., 2014 [87]	USA	Case report	G	F	11	Intermittent abdominal pain, vomiting, diarrhea, duodenal peptic ulcer	Gastrin > 100,000 pg/mL	-	US + PET CT + MR	Tail + liver mts	-	Distal pancreatectomy + liver transplant	-	Asymptomatic after transplant and chemotherapy
Dall’igna et al., 2010 [88]	Italy	Original research	G	F	14	Zollinger–Ellison syndrome	-	-	-	Head + liver mts	2	Biopsy + octreotide	-	Alive with stable disease at 16 months
Schettini et al., 2009 [89]	Brazil	Case report	G	M	11	Epigastric pain, retching, vomiting, diarrhea, gastric ulcer	Gastrin—1000 pg/mL	-	CT	Head + liver mts	1.5	Enucleation	-	No recurrence
Gurevich et al., 2003 [90]	Russia	Original research	G	M	15	Zollinger–Ellison syndrome	Gastrin—260 pg/mL	-	-	Head + liver mts	4.5	-	-	-
Wamsteker et al., 2003 [91]	USA	Original research	G	F	15	Asymptomatic	Gastrin—169 pg/mL	MEN1	EUS	Head, uncinate process, body, tail	0.5–0.7	-	-	EUS surveillance
Eire et al., 1996 [92]	Spain	Case report	G	F	14	Suffered stomach, diarrhea	Gastrin—1500 pg/mL	-	US	Head	-	Partial gastrectomy and subtotal pancreaticoduodenectomy	-	No recurrence

G—gastrinoma, M—male, F—female, US—ultrasound, EUS—endoscopic ultrasound, MR—magnetic resonance, CT—computed tomography, PET—positron emission tomography, MEN1—multiple endocrine neoplasia type 1, mts—metastases.

**Table 3:** Articles included in the systematic review—glucagonomas.

Study	Country	Type of Study	Type of Tumor	Patient’s Gender	Patient’s Age (Years)	Clinical Presentation	Laboratory Findings	Genetic Analysis	Radiological Modality	Tumor Location	Tumor Size (cm)	Type of Treatment	Histopathology	Outcome
Luber et al., 2016 [9]	USA	Case report	GL	F	15	Persistent, pruritic, and painful rash; hair loss; brittle nails; diarrhea; poor weight gain	Glucagon—3076 pg/mL	-	CT	Uncinate process	3.4 × 4.3 × 2.9	Whipple procedure	NET G1	No recurrence
van den Akker et al., 2012 [49]	Belgium	Original research	GL	M	12	-	-	-	-	-	4	Distal pancreatectomy	-	No recurrence
van Beek et al., 2004 [93]	Netherlands	Case report	GL	M	16	No complaints	Glucagon—145 pmol/L	MEN1	Angiography + MR	Body	2	Subtotal pancreatectomy	-	No recurrence

GL—glucagonoma, M—male, F—female, MR—magnetic resonance, CT—computed tomography, NET—neuroendocrine tumor, G—gradus, MEN1—multiple endocrine neoplasia type 1.

**Table 4:** Articles included in the systematic review—VIPomas.

Study	Country	Type of Study	Type of Tumor	Patient’s Gender	Patient’s Age (Years)	Clinical Presentation	Laboratory Findings	Genetic Analysis	Radiological Modality	Tumor Location	Tumor Size (cm)	Type of Treatment	Histopathology	Outcome
Bonilla Gonzalez et al., 2021 [94]	Colombia	Case report	V	F	14	Diarrhea, abdominal pain, vomiting, hypoxia, arthralgia, myalgia	VIP—91.2 pmol/L	-	Scintigraphy + MR	Body	2 × 1.6, 1 × 0.9	Distal pancreatectomy	NET G2	No recurrence
Yeh et al., 2020 [11]	Taiwan	Case report	V	F	7	Blood-tinged and mucoid diarrhea, poor appetite, general weakness	VIP—743.82 pg/mL	-	CT + MRCP	Body	-	Partial pancreatectomy	-	Therapy: everolimus + octreotide
Acosta-Gualandri et al., 2019 [95]	Canada	Case report	V	F	13	Profuse watery diarrhea, nausea, vomiting, dehydration, abdominal pain	VIP—1105 pg/mL	MEN1	CT + MR	Tail	4.5	Distal pancreatectomy	NET G1	Withdrawal of symptoms
Bourcier et al., 2013 [96]	USA	Case report	V	M	12	Dehydration, diarrhea, fainting, flushing	VIP—134.5 pg/mL	-	CT + MR	Tail	1.5 × 1.0 × 0.8 + liver mts	Distal pancreatectomy	-	Therapy
Masulovic et al., 2012 [97]	Serbia	Case report	V	M	15	Chronic diarrhea, abdominal pain, nausea, vomiting	-	MEN1	CT + MR	Head, body	3.8, 1.2	Whipple procedure + enucleation	-	No recurrence
Brenner et al., 1986 [98]	USA	Case report	V	F	15	Diarrhea, vomiting	VIP—2150 pg/mL	-	ERCP	Body, tail	6 × 5	Distal pancreatectomy (85%)	-	No recurrence

V—vipoma, M—male, F—female, MR—magnetic resonance, CT—computed tomography, MRCP—magnetic resonance cholangiopancreatography, ERCP—endoscopic retrograde cholangiopancreatography, VIP—vasoactive intestinal peptide, NET—neuroendocrine tumor, G—gradus, MEN1—multiple endocrine neoplasia type 1, mts—metastases.

**Table 5:** Articles included in the systematic review—mixed functional pancreatic tumors.

Study	Country	Type of Study	Type of Tumor	Patient’s Gender	Patient’s Age (Years)	Clinical Presentation	Laboratory Findings	Genetic Analysis	Radiological Modality	Tumor Location	Tumor Size (cm)	Type of Treatment	Histopathology	Outcome
Petriczko et al., 2022 [99]	Poland	Case report	I/GL	M	17	Hypoglycemia, epileptic seizures	Glucose—1.94 mmol/L, insulin—5.13 uIU/mL	MEN1	US + EUS + CT	Head + body + tail	0.7, 1.1, 1.2	Surgical resection + reoperation	NET G1/G2	Glycemic normalization
Erichsen et al., 2020 [100]	Denmark	Case report	I/GL	F	14	Unrecognized hypoglycemia symptoms	Glucose—2.5 mmol/L, p-insulin 158 pmol/L, glucagon—1000 pmol/L	MEN1	EUS + PET CT + MR	Head + tail + uncinate process	1	Surgical resection x4	-	-
Winston et al., 2014 [101]	Canada	Case report	I/GL	M	14	Abnormal behavior	Glucose—2.1 mmol/L, insulin—82.9 pmol/L	MEN1	US + MR	Body + tail	2.9 × 3.2 × 2.4, 2.5 × 1.5 × 2	Sub-total pancreatectomy	-	Glycemic normalization

I—insulinoma, GL—glucagonoma, M—male, F—female, US—ultrasound, EUS—endoscopic ultrasound, MR—magnetic resonance, CT—computed tomography, PET—positron emission tomography, NET—neuroendocrine tumor, G—gradus, MEN1—multiple endocrine neoplasia type 1, mts—metastases.

#### 3.2.1. Insulinomas

Gender data were available for 134 participants, of whom 78 (58.2%) were male and 56 (41.8%) were female. The median age was 12 years (range 30 h to 17 years). A total of 45 cases were reported in childhood (<10 years), while 94 cases were reported in adolescence (≥10 years). Although clinical presentations vary from case to case, in all but one case (incidental diagnosis), either recurrent hypoglycemia, neuroglycopenic symptoms, or adrenergic symptoms in response to hypoglycemia were reported. Symptoms usually occurred during fasting, exercise, or at night, and usually improved after eating. In several cases, clinical presentations were misinterpreted as behavioral or psychiatric problems. In most cases, where glucose and insulin levels were recorded, the levels were abnormal, with serum insulin ≥ 3 μU/mL (or ≥18 pmol/L) and glucose < 55 mg/dL (3.0 mmol/L). Genetic testing revealed that insulinoma was associated with MEN1 in 49 cases (35.3%). According to available data, the male-to-female ratio in MEN1 cases was 21:15, while in sporadic cases it was 48:28 (*p* = 0.62). The mean age was 11.65 ± 3.22 and 11.19 ± 4.51 (*p* = 0.53), respectively. There was also no difference in tumor grade (*p* = 0.49), nor in the type of surgical treatment (enucleation vs. some form of resection) (*p* = 0.29). In relation to the available data for the location of insulinoma, 95 (81.9%) were in single location (solitary), while 21 (18.1%) were in multiple. Of the cases with multiple locations, 13 (61.9%) were associated with MEN1, while only 21 (22.1%) of the single ones were associated with MEN1 (*p* = 0.0003). For 144 tumors, the location could be determined, for either sporadic or multiple tumors. Seven (4.9%) of them were localized in the unicinate process, 37 (25.7%) in the head, 8 (5.6%) in the neck, 34 (23.6%) in the body, and 58 (40.2%) in the tail. Of the cases with multiple locations, the most frequent combination was body/tail (6 cases), followed by head/body/tail (5 cases). Metastases were noted in four cases, predominantly hepatic. The dominant diagnostic radiological methods used were non-invasive (combination of abdominal US, CT, PET-CT, MRI). Endoscopic ultrasound was used in 24 cases. ASVS was used in four cases. The mean size of all recorded tumors from the available data was 1.64 cm, while the median was 1.5 cm (range 0.2–12.5 cm). A total of 41 were less than 1 cm, 84 were 1–2 cm, 26 were 2–3 cm, and 10 were larger than 3 cm. Regarding the type of surgical treatment, tumor enucleation was performed in 68 cases, while some form of resection was performed in 71 cases. A laparoscopic approach was used in nine cases, while a robotic approach was used in three cases. In the available data from 43 patients, 20 tumors had histological pNET grade 1, 25 tumors had histological grade 2, and only one had grade 3 in metastases. Reoperation was performed in eight cases (of which enucleation was performed as the first surgery in six cases), while four children died. One patient died of an unrelated cause. The main characteristics of the insulinoma studies included in this systematic review are presented in Table 1.

#### 3.2.2. Gastrinomas

Of the 12 recorded cases of gastrinomas, 6 (50%) were male, and 6 (50%) were female. The median age was 13 years (range 6 to 17 years). There were two under the age of 10. The clinical presentation in almost all patients was dominated by peptic ulcer, epigastric pain, and diarrhea. Only one patient was asymptomatic. In all cases for which data were available, gastrin levels were elevated. In six patients, levels ranged between 100 and 1000 pg/mL, while in five, they were ≥1000 pg/mL. Genetic testing confirmed MEN1 in four patients (33%). Noninvasive radiological examinations such as abdominal US, CT, PET-CT, and MRI dominated. EUS was performed in only one patient. In relation to the available data for the location of gastrinomas, eight were in a single location (solitary), while two were in multiple locations. Both multiples were associated with MEN1. Most of them were localized in the head of the pancreas (*n* = 7). Liver metastases were noted in five patients. The mean size of all recorded tumors from the available data was 1.9 cm, while the median was 1.5 cm (range 0.2–4.5 cm). In two patients, the tumor was smaller than 1 cm, while in four it was ≥2 cm. Regarding the type of surgical treatment, resections predominated, one of which was performed laparoscopically. No tumor was histologically graded. One patient with MEN1 underwent reoperation. No child died during the follow-up period. The main characteristics of the gastrinomas studies included in this systematic review are shown in Table 2.

#### 3.2.3. Glucagonomas

A systematic review identified three patients with glucagonoma. Two were male and one was female. The mean age was 14.3 years, with no patient younger than 10 years. In one patient, there were documented symptoms of persistent, pruritic, and painful rash; hair loss; brittle nails; diarrhea; and poor weight gain. Glucagon levels were documented in two patients. One patient was associated with MEN1. As for radiological diagnostics, one patient underwent a CT scan, and the other underwent an angiography and an MRI. The mean tumor size was 3.4 cm. One was located in the uncinate process and the other in the body. All cases underwent some form of pancreatic resection. One tumor was histologically grade 1. No recurrence was noted in any patient during follow-up. The main characteristics of the glucagonoma studies included in this systematic review are shown in Table 3.

#### 3.2.4. VIPomas

Of the six recorded cases of VIPoma, four (66.7%) occurred in females and two (33.3%) in males. The mean age was 12.7 years. Only one patient was younger than 10 years. The dominant symptoms experienced by most patients were watery diarrhea, mild abdominal pain, and symptoms associated with dehydration, such as lethargy, nausea, vomiting, and muscle weakness. VIP levels were significantly elevated in more than half of the cases. Genetic testing in two patients confirmed MEN1. MRI and CT were the dominant imaging modalities in most patients. Four tumors appeared solitary, while two were multiple. Of the solitary ones, two appeared in the body and two in the tail. Multiples appeared in the head/body and body/tail combinations. Liver metastases were noted in one patient. The mean size of all recorded tumors from the available data was 3.1 cm. Regarding the type of surgical treatment, distal pancreatectomy was performed in five of six cases (83.3%), while enucleation with the Whipple procedure was performed in one case. Of the available data, one patient had a histological pNET grade 1 tumor, and the other had a pNET grade 2 tumor. Although pharmacological therapy continued in some patients, no deaths or the need for reoperation were recorded during the follow-up period. The main characteristics of the VIPoma studies included in this systematic review are shown in Table 4.

#### 3.2.5. Mixed Functional pNETs

Regarding mixed pancreatic functional tumors in children and adolescents, three cases of coexistence of glucagonomas and insulinomas have been reported, in one female and two males, aged 14 and 17 years. The clinical presentation in all three cases was dominated by neuroglycopenic symptoms. All three cases were related to MEN1 and were multiples, with a mean size of 1.6 cm. The tumors were in all locations of the pancreas. The main characteristics of the mixed functional pNET studies included in this systematic review are shown in Table 5.

## 4. Discussion

Of the included studies, 71 were insulinomas, 10 were gastrinomas, 3 were glucagonomas, 6 were VIPomas, and 3 were mixed-function pNETs. Of the available data, 57% were male and 43% were female. The median age was 12 years. A total of 48 cases were reported in childhood, while 115 cases were reported in adolescence. Almost all patients presented with symptoms appropriate to the type of tumor. A significant proportion of tumors were associated with MEN1. In almost all patients, the symptomatology was accompanied by elevated levels of specific hormones. US, CT, PET-CT, MRI, and EUS were the dominant imaging modalities. Surgical approaches and types of resections, depending on the type, association with the syndrome, location, and size of the tumor, were quite heterogeneous. Tumor grades G1 and G2 were almost equally represented. There was no recurrence in most patients.

The incidence of insulinomas estimated from case series ranged from 0.13 to 0.4 cases per 100,000 person-years, with a median age at diagnosis of approximately 47–56 years, with a slight female predominance, in contrast to our results in childhood and adolescence, where a male predominance was found [102,103,104]. Compared to our results, where insulinoma was associated with MEN1 in 35.3% of cases, previous studies estimated the association at 4 to 10% [105,106]. We believe that the increased association can be attributed to advances in diagnostic capabilities and increased genetic testing, increased awareness among clinicians, and the development of screening protocols in pediatric endocrinology. It is also possible that insulinomas in the context of MEN1 are manifesting earlier in life, possibly because of genetic or environmental factors that influence tumor development at a younger age. As in adults, the most common clinical manifestation of insulinoma in children is neuroglycopenic symptoms, which may or may not be preceded by sympathoadrenal symptoms. Also, a significant proportion of adults are misdiagnosed as having neurological or psychiatric disorders, as was noted in our review [107,108]. In a case series, Melikyan et al. found that children are more likely to develop neuroglycopenic symptoms, and up to half of them were misdiagnosed [6]. Initial misdiagnosis should be attributed to nonspecific symptoms that may mimic various neurological or psychiatric conditions, intermittent and variable presentation, and lack of awareness by clinicians due to the rarity of the tumor itself. Our review found that in children and adolescents, the majority of insulinomas are solitary (81.9%), as has been reported in previous studies [109,110]. Also, as in previous studies, multiple tumors were more frequently associated with MEN1 [104]. There is no doubt that in MEN1, the genetic defect affects all cells carrying the mutation, leading to a high probability of multiple tumors (multicentricity) within the same organ. As noted here, insulinomas have also been previously reported in the series to have an average size of 1.5 cm [111]. This size likely represents the clinical threshold at which the tumor begins to cause significant symptoms and becomes detectable by conventional imaging modalities, such as abdominal US, CT, and MRI. Regarding radiological diagnostics, after laboratory indicators of hyperinsulinism hypoglycemia and suspicion of insulinoma, non-invasive imaging modalities such as abdominal US, CT, PET-CT, or MRI are usually resorted to [112,113,114]. EUS provides high-resolution imaging of the pancreas and can detect lesions as small as 2 mm in diameter, improving sensitivity to nearly 100% when combined with noninvasive imaging modalities. EUS’s combination of high-resolution imaging, proximity to the pancreas, and capability for tissue sampling makes it particularly valuable for detecting and localizing insulinomas, especially when other non-invasive imaging modalities are inconclusive or insufficient for small lesion detection [115,116]. If evidence of disease is lacking in previous radiological imaging modalities, highly specialized localization assessments such as arterial stimulation with venous sampling (ASVS) can be performed [104,117]. EUS was performed in 24 patients in our cases, while ASVS was performed in four. For patients with localized disease, surgical removal of the insulinoma is the treatment of choice. Surgical approaches to insulinoma removal include tumor enucleation and parenchymal-sparing resection of the pancreas. Guidelines suggest enucleation of insulinomas < 2 cm and located > 2 to 3 mm from the main pancreatic duct. Traditional resection (pancreaticoduodenectomy, distal pancreatectomy, or total pancreatectomy) with appropriate lymphadenectomy, rather than parenchymal-sparing resection or enucleation, is recommended for patients with functional pNET > 2 cm, which has a higher risk of malignancy and potential for lymph node disease, and/or those bordering the pancreatic duct [118,119,120,121]. As in our review (8.8%), according to Crippa et al., 8.5% of patients who initially underwent insulinoma enucleation required reoperation [122]. Enucleation may target the most obvious tumor, but undetected additional tumors may be missed. Some insulinomas are very small or located in difficult anatomic locations, which may evade intraoperative detection by palpation or standard imaging. Even when a tumor is identified, enucleation may not provide clear margins, leading to persistent or recurrent hyperinsulinemia. This emphasizes the importance of meticulous preoperative planning and intraoperative exploration to reduce the likelihood of residual disease. It is recommended that insulinoma reoperation be performed only by an experienced surgeon, with previous sophisticated endocrinological and radiological support. Blind resection of the pancreas should not be performed if localization studies fail to detect a tumor. As in our cases, the liver is the most common site of metastasis. In selected cases, they can be resected together with the primary tumor [123,124,125]. It should certainly be emphasized that when observing the cases of the included studies, a heterogeneous surgical approach is observed, with a significant proportion not following the guidelines, which should certainly be corrected in the future by surgeons who will encounter this pathology. The heterogeneity of surgical approaches to insulinoma likely stems from several factors. Precise preoperative localization can sometimes be challenging, which influences the choice of surgical approach. Although guidelines provide a framework, they are based on the available evidence, which is still evolving. Individual surgeons’ experience with specific procedures and variations in healthcare systems, access to technology, and training may influence practice patterns. No evidence-based guidelines are available to guide follow-up after surgical treatment of insulinoma. Recurrences are more common in patients with MEN1. The higher recurrence rate of insulinomas in MEN1 patients is primarily due to the genetic predisposition to multiple and recurrent tumors, the presence of multicentric disease, and ongoing pancreatic islet cell hyperplasia. This necessitates careful long-term monitoring and often a more comprehensive treatment approach in MEN1-associated insulinomas. In a multicenter study by Crippa et al., 3% of patients experienced disease recurrence during a median follow-up of 65 months [111,122].

Zollinger–Ellison syndrome (ZES) is caused by gastrin secretion by duodenal or pancreatic neuroendocrine tumors (gastrinomas). The reported incidence is between 0.5 and 2 per million population, with most diagnoses occurring between the ages of 20 and 50 years, with a slightly higher incidence in males [118,126,127]. In our review, the proportion of females and males was equal. Similar to our review, it has previously been reported that approximately 80 percent of gastrinomas are sporadic, and that 20 to 30 percent occur in association with MEN1 [128,129]. As in our cases, the clinical presentation is dominated by peptic ulcer, diarrhea, heartburn, epigastric pain, and complications caused by acid hypersecretion [130,131]. It is important to note that ulcers in the context of ZES are more prone to refractoriness to proton pump inhibitor therapy and recurrence compared to in patients with sporadic ulcer disease. This is because elevated serum gastrin levels stimulate continued, unregulated acid secretion by gastric parietal cells, leading to persistent hyperacidity. Sometimes higher doses of proton pump inhibitors are required, and sometimes even then, complete suppression may not be achieved [132]. The diagnosis is not straightforward, as indicated by the fact that the median time from symptom onset to diagnosis is >5 years. Elevated serum gastrin levels, combined with a history of current or recent peptic ulcer disease and with diarrhea that is responsive to proton pump inhibitors (PPIs), support the diagnosis of ZES [133]. A serum gastrin value greater than 10 times the upper limit of normal (1000 pg/mL) in the presence of a gastric pH below 2 is diagnostic of ZES [134]. In our review, five patients had gastrin levels ≥ 1000 pg/mL. Although gastric pH assessment is classically required for a diagnosis of ZES, case series, as in our review, indicate that gastric acidity assessment is underutilized [127,129]. This is likely due to the technical complexity, limited availability, patient discomfort, and effectiveness of alternative diagnostic tests. Although pharmacologic acid suppression therapy is the standard of care for most patients with gastrinomas, many patients with localized sporadic gastrinomas are candidates for surgical treatment in addition to pharmacologic therapy. A total of 80% of curable gastrinomas lie within the gastrinomas triangle, which consists of the head of the pancreas and the duodenal layer [134,135]. As many as seven patients from our review had tumor localization exclusively in the head of the pancreas. Early surgical treatment, even with incomplete gastrinomas tissue resection, seems to have a favorable effect on the course of the disease. In general, the cure rate of sporadic, non-metastatic gastrinomas after one and five years is 60 percent and 30 to 40 percent, respectively [136,137,138]. Guidelines suggest traditional resection of all pancreatic gastrinomas [134,139,140]. Compared with other pancreatic NETs, minimally invasive surgery is controversial in gastrinomas, due to the need for more extensive exploration of the gastrioma triangle, lymphadenectomy, and duodenectomy in many cases. These additional procedures can be technically challenging to perform, and the need for thorough exploration and resection to ensure complete tumor removal and address potential lymph node metastases makes the approach more complex and debated among surgeons [130]. Metastatic disease is the most common cause of morbidity and mortality in patients with gastrinomas. The higher incidence of liver metastases in gastrinomas is mainly due to early vascular invasion into the portal vein, aggressive growth characteristics, and a tendency to present at a more advanced stage [141,142]. Liver metastases were recorded in as many as five patients in our review, but no deaths were recorded during follow-up. There is limited evidence from which to make recommendations for follow-up after resection of a gastrinoma.

The incidence of glucagonomas is estimated at 0.01 to 0.1 per 1,000,000. They usually present around the age of 50 [143,144,145]. Glucagonomas are generally slow-growing but are usually advanced by the time of diagnosis. The most common symptoms related to glucagon are weight loss, necrolytic migratory erythema, and chronic diarrhea, which were observed and documented in one patient in our review [146,147]. Plasma glucagon levels are usually elevated 10- to 20-fold (>500 pg/mL) in patients with glucagonoma (normally <50 pg/mL). In the differential diagnosis, elevated serum glucagon can also be caused by hypoglycemia, fasting, trauma, sepsis, acute pancreatitis, abdominal surgery, Cushing’s syndrome, and renal and hepatic failure [143,145]. Most of them are sporadic, and one-fifth of them are associated with MEN1. They are usually large (>3 cm) and usually occur in the distal part of the pancreas. The combination of non-specific early symptoms, slow tumor growth, and lack of early biochemical warning signs results in glucagonomas being larger at diagnosis compared to other functional pancreatic neuroendocrine tumors [143,144]. Of the three patients included in the systematic review, one was associated with MEN1. Given the size of the glucagonoma, non-invasive radiological methods such as CT, PET-CT, and MR are sufficient to detect the tumor, which was also documented in our cases [148,149]. In our review, apart from the fact that all tumors were solitary, the tumor sizes were larger than 2 cm and were detected in the uncinate process and body by non-invasive imaging methods. The type of pancreatic resection is dictated by the location and extent of the tumor at the time of the procedure. Glucagonomas are usually located in the body or tail of the pancreas and can be treated with distal pancreatectomy [150,151].

VIPomas are diagnosed in approximately 1 in a million people per year, usually between the ages of 30 and 50 years [152,153]. Unlike the pancreas, VIP-secreting tumors usually arise in the sympathetic ganglia and adrenal glands [10]. As noted in our systematic review, the literature to date suggest that symptomatic pancreatic VIPomas are usually solitary, larger than 3 cm in diameter, and most commonly occur in the tail of the pancreas [154,155]. Regarding the specific symptomatology associated with VIPomas, most patients with a VIPoma have VIPoma syndrome, also called pancreatic cholera syndrome; Verner–Morrison syndrome; and watery diarrhea, hypokalemia, and hypochlorhydria or achlorhydria (WDHA) syndrome [156]. In addition to radiological imaging (usually CT and MRI), the diagnosis is based on elevated serum levels of vasoactive intestinal polypeptide. In one observational series of 52 patients with VIPoma, the median VIP level was 630 pg/mL, which is similar to our results [153,157]. Given the dominant location and size of the tumor, primary tumors are usually surgically treated with distal pancreatectomy, as noted in our cases [158]. Before resection, all patients with VIPoma require correction of dehydration, hypokalemia, and other metabolic abnormalities, and preoperative administration of octreotide can reduce circulating VIP levels [159]. There are limited data on the optimal posttreatment surveillance following resection of a VIPoma [140].

Coexistence of glucagonomas and insulinomas has been reported in adults, mostly with MEN1. As in our cases, neuroglycopenic symptoms dominated in all the cases described so far. In all cases described so far, a wide range of tumor sizes, from 0.2 to 4 cm, in different locations has been reported [160]. The relationship between insulinomas and glucagonomas is still unclear with respect to the occurrence of mixed tumors. It is possible that prolonged hyperinsulinemia and hypoglycemia cause secondary α-cell hyperplasia, leading to neoplasia, but it is not clear why this occurs in such a small number of patients. Another possibility is that malignant insulinomas transform into a different functional tumor [161,162,163]. Further research is needed to accept or reject possible hypotheses.

According to our systematic review, where we did not record a single case of somatostatinoma in childhood or adolescence, with an incidence of 1 in 40 million people, somatostatinoma is known to be the rarest pNET. The molecular pathways leading to somatostatinoma may likely require accumulated genetic mutations or environmental exposures, delaying tumor development until adulthood. Excessive secretion of somatostatin produces a specific syndrome that includes steatorrhea, mild diabetes, and cholelithiasis. Most somatostatinomas are solitary, are located in the head of the pancreas or duodenum, and can be treated with pancreatoduodenectomy [164,165].

In general, genetic screening, i.e., targeted mutation analysis, for functional pancreatic tumors should be tailored based on a combination of factors, including the functional tumor type, the presence of multiple tumors, and the patient’s age at presentation. Multiple tumors may be suggestive of inherited syndromes. Younger patients presenting with pancreatic tumors are more likely to have inherited or germline mutations. Cases with early onset and multiplicity often warrant comprehensive germline genetic testing to detect inherited syndromes, which may influence treatment strategies and screening for family members [166].

The goal of long-term follow-up of patients who have undergone surgical treatment for a functional pancreatic tumor should be the early detection of recurrence or the development of a new tumor or associated endocrine tumors to optimize outcomes and reduce morbidity. Regular measurement of relevant hormones depending on the tumor type, cross-sectional imaging (MRI) at regular intervals, and EUS if minor lesions are suspected are necessary. The psychological component of the impact of long-term treatment in children should not be neglected. It is also necessary to ensure a smooth transition to adult care with continuity of the follow-up protocol.

Advances in diagnostic tools such as multi-analytical circulating biomarkers and liquid biopsy (circulating tumor cells, circulating tumor DNA, microRNAs, exosomes, protein-based biomarkers) are certainly changing the way we detect and monitor neuroendocrine tumors. By analyzing multiple biomarkers simultaneously (multi-analytical approach), clinicians can improve the sensitivity and specificity of detection, helping to identify tumors at earlier stages and monitor response to treatment. The advantages are primarily non-invasiveness, real-time monitoring, and early detection [167,168,169,170]. There is no doubt that the above detection and monitoring methods will be increasingly used in the future, especially in children and adolescents.

In the context of limited existing evidence, adapting the currently published guidelines for the management of functional pancreatic tumors to pediatric patients requires a careful, multidisciplinary, and individualized approach. We strongly recommend collaborating with centers of excellence that have addressed similar cases or have established registries. Professional societies will certainly need to establish consensus panels in due course to formulate interim guidelines based on the best available evidence, clinical experience, and case reports.

## 5. Conclusions

Although functional pancreatic neuroendocrine tumors in children are extremely rare, early suspicion based on specific clinical symptomatology is essential for timely diagnosis. Accurate localization and size based on modern radiological diagnostics, accompanied by biochemical and genetic testing, are essential for optimal management, which must be multidisciplinary and carried out by a pediatric endocrinologist, oncologist, radiologist, surgeon, and pathologist. Adequate surgical resection offers the best chance of cure, with the lowest risk of recurrence. Due to the rarity of these tumors in children, additional multicenter registries and studies are needed in the future to better understand tumor behavior, optimal treatment, and outcomes.

## Data Availability

The data that support the findings of this study are available upon request from the corresponding author.

## Supplementary Materials

The following supporting information can be downloaded at: https://www.mdpi.com/article/10.3390/diagnostics15172176/s1, Table S1: Methodological quality of included studies according to JBI Critical Appraisal Checklist for Case Report and Case Series. S1. PRISMA 2020 Checklist.

## Abbreviations

The following abbreviations are used in this manuscript:

pNETs	Pancreatic neuroendocrine tumors
MEN1	Multiple endocrine neoplasia type 1
VHL	Von Hippel–Lindau disease
NF1	Neurofibromatosis type 1
TSC	Tuberous sclerosis complex
ZES	Zollinger–Ellison syndrome
NME	Necrolytic migratory erythema
VIP	Vasoactive intestinal peptide
US	Ultrasound
EUS	Endoscopic ultrasound
MR	Magnetic resonance
CT	Computed tomography
ASVS	Arterial calcium stimulation with venous sampling
PET	Positron emission tomography
NET	Neuroendocrine tumor
G	Gradus
hPTH	Hyperparathyroidism
hPRL	Hyperprolactinemia
DM	Diabetes mellitus
Mts	Metastases
MRCP	Magnetic resonance cholangiopancreatography
ERCP	Endoscopic retrograde cholangiopancreatography
PPIs	Proton pump inhibitors
